# Lingual Tonsillectomy for Resistant Obstructive Sleep Apnea in Children with Down Syndrome: A Systematic Review and Meta-Analysis

**DOI:** 10.1177/00034894251407787

**Published:** 2026-01-03

**Authors:** Emma Finnegan, Leticia Campos, Anna Mulcahy, Jaime Doody

**Affiliations:** 1 School of Medicine, Trinity College Dublin, Ireland; 2 Universidade de Ribeirão Preto, São Paulo, Brazil; 3 Division of Otolaryngology, Stony Brook School of Medicine, NY, USA

**Keywords:** obstructive sleep apnea, down syndrome, lingual tonsillectomy, adenotonsillectomy, airway

## Abstract

**Introduction:**

Obstructive sleep apnea (OSA) affects many children, particularly those with Down syndrome (DS), with prevalence estimates ranging from 50% to 100%. Although adenotonsillectomy is the traditional first-line treatment, individuals with DS frequently have residual OSA (rOSA) because to their complex airway architecture. Lingual tonsil hypertrophy has been found as a significant risk factor for rOSA; however, the role of lingual tonsillectomy (LT) in this population is uncertain.

**Methods:**

We conducted a systematic review and meta-analysis according to PRISMA and Cochrane guidelines, registered with PROSPERO (CRD42024552955). We included studies of pediatric patients (≤18 years) with DS and rOSA who underwent LT. Primary outcomes were changes in apnea-hypopnea index (AHI), obstructive AHI (OAHI), and oxygen nadir (O₂). Secondary outcomes included postoperative complications.

**Results:**

Three retrospective case series comprising 68 patients met the inclusion criteria. Pooled analysis demonstrated a mean improvement in oxygen nadir of 4.58% (95% CI: 2.73-6.43, *I*^2^ = 0%). AHI and OAHI scores were decreased by −2.97 (95% CI: −8.88 to 2.94, *I*^2^ = 24%) and −8.20 (95% CI: −13.46 to −2.94, *I*^2^ = 0%) respectively. Complication rates were low but inconsistently reported.

**Conclusion:**

Lingual tonsillectomy appears effective in improving OSA severity in children with DS. While promising, outcome improvements may be less pronounced than in the general population, likely due to complex anatomical and physiological factors unique to this group. Standardized outcome measures and prospective studies are needed to better guide management in this high-risk group.

## Introduction

Obstructive sleep apnea (OSA) is a condition characterized by the partial or complete collapse of the upper airways during sleep,^1^ leading to episodes of hypopnea and apnea.^2^ Pediatric OSA affects an estimated 1% to 5.8% of children^3^ and is associated with behavioral and cognitive challenges, reduced quality of life, and impaired development.^3,4^ The prevalence of OSA is significantly higher in children with Down syndrome, with childhood estimates ranging from 50% to 100%.^5-11^ The increased prevalence in individuals with Down syndrome is likely linked to anatomical and physiological abnormalities commonly found in this population. These include obesity, midfacial hypoplasia, relative macroglossia, and muscular hypotonia, which contribute to airway collapsibility and exacerbate sleep-disordered breathing.^2,12,13^

Breathing disruptions in OSA, particularly in children with Down syndrome, lead to intermittent hypoxia, recurrent nocturnal arousals, increased sympathetic nerve activity, and fragmented sleep.^4,5^ Adenotonsillectomy is widely recognized as the first-line treatment for pediatric OSA.^14,15^ In certain cases, adenotonsillotomy – a less invasive, tissue-sparing alternative – may be considered, offering benefits such as reduced postoperative pain and faster recovery.^16-18^ However, OSA often persists following these interventions, necessitating additional treatments.^19^ Tonsillectomy is additionally associated with a relatively high rate of regrowth.^20,21^

Lingual tonsil hypertrophy has been identified as a significant cause of residual OSA in children post-adenotonsillectomy.^22-24^ Lingual tonsillectomy as a procedure involves transoral removal or ablation of lymphoid tissue of the lingual tonsils located at the base of the tongue, which form part of Waldeyer’s ring.^25^ As a result, lingual tonsillectomy has been increasingly utilized to address persistent pediatric OSA, with numerous studies and meta-analyses demonstrating its efficacy.^26-29^

Despite the significant incidence of persistent OSA in children with Down syndrome, there is currently no meta-analysis or consensus on the use of lingual tonsillectomy within this specific cohort. This systematic review and meta-analysis aims to evaluate the effectiveness of lingual tonsillectomy in managing resistant obstructive sleep apnea (rOSA) in children with Down syndrome, addressing a critical gap in the literature. By focusing on this unique population, the study seeks to provide evidence-based guidance for the treatment of rOSA in children with Down syndrome.

## Materials and Methods

This systematic review and meta-analysis was conducted under the Cochrane Collaboration Handbook for Systematic Reviews of Interventions^30^ and the Preferred Reporting Items for Systematic Reviews and Meta-Analysis (PRISMA) statement guidelines.^31^ The protocol for this study was prospectively registered in the International Prospective Register of Systematic Reviews (PROSPERO), under the registration number CRD42024552955.

### Eligibility Criteria

Inclusion in this meta-analysis was limited to studies meeting all the following criteria: (1) randomized controlled trials, cohort studies, case-control studies, or case series, (2) evaluating the efficacy of lingual tonsillectomy, (3) conducted in a pediatric population (≤18 years) with residual obstructive sleep apnea (rOSA) following adenotonsillectomy, and (4) involving patients with Down syndrome. Only studies reporting on clinical outcomes of interest were included. We excluded patients undergoing concurrent extensive surgeries involving the base of the tongue, however, patients receiving surgeries of the nasal passage, epiglottis, or revision adenotonsillectomies were included. We excluded studies that (1) were single-patient case reports, (2) assessed an adult population, (3) included additional tongue base resections, such as midline glossectomies (4) did not specify outcomes for individuals with Down syndrome within a pediatric population, and (5) were not available in English.

### Search Strategy and Data Extraction

We systematically searched PubMed, Scopus, and Cochrane Central Register of Controlled Trials with key search terms, including “obstructive sleep apnea” “pediatric” and “Down syndrome.” Full search strategy details are available in Supplemental Table 2. The references from all included studies were also searched manually for any additional studies. Two authors (L.R.C. and E.F.) independently extracted the data following predefined search criteria and quality assessment.

### Endpoints and Subanalyses

Clinical measures were reported as provided by the individual studies. Primary outcome measures included changes in measurements of OSA severity, including apnea-hypopnea index (AHI), obstructive apnea-hypopnea index (OAHI) and minimum oxygen saturation (O₂ nadir). Secondary outcomes included complications in these individuals such as post-operative bleeding, dysphagia, poor oral intake, pain, or airway obstruction. Where data relating to outcomes was not readily available in texts, study authors were contacted.

### Quality Assessment

Nonrandomized studies were appraised with the Risk of Bias in Nonrandomized Studies of Interventions (ROBINS-I) tool.^32^ This was performed independently by E.F. and L.C. In the case of discordant results, a third author A.M. was involved to come to a final decision. Publication bias was investigated by funnel-plot analysis of point estimates according to study weights and by Egger’s regression test.

### Statistical Analysis

A single-arm meta-analysis was performed to estimate the mean effect size for each outcome, with 95% confidence intervals (CI) as a measure of variability. A random-effects model was applied to account for heterogeneity among studies. Data synthesis and pooling of means were conducted using the “meta” package in R.^33^ Heterogeneity among studies was assessed using the Cochran *Q* test and *I*² statistic. We utilized the Cochrane pre-defined threshold for interpreting heterogeneity, with 0 to 40% representing low heterogeneity, 30% to 60% moderate, 50% to 90% substantial, and 75% to 100% considerable heterogeneity. All statistical analysis was performed using The R Project for Statistical Computing, Vienna, Austria (Version 4.3.2).

## Results

### Study Selection and Characteristics

**Figure 1.**
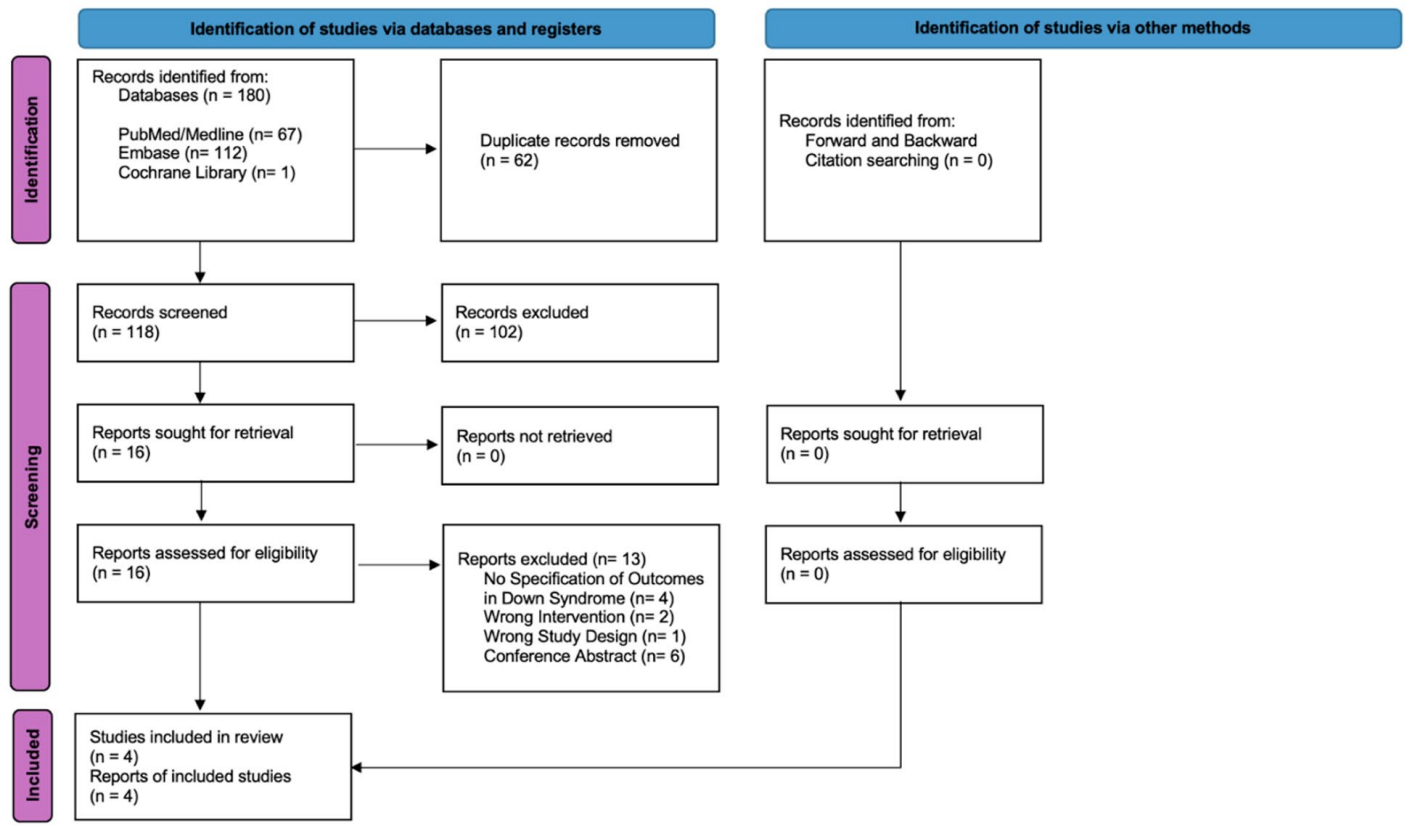
PRISMA flow diagram of study screening and selection.

As shown in Figure 1, the initial search retrieved 180 results. After removing duplicate records and ineligible studies, 16 studies were evaluated based on full texts against the inclusion criteria. Following a thorough full-text review, 3 studies met the inclusion criteria and were included in the final analysis.^29,34,35^ There were a total of 68 patients across the 3 studies. All 3 studies were retrospective case series, each assigned an evidence-level rating of 4.

The mean age of all patients was 10.34 years (SD 4.08). Women made up 47.06% of the cohort (n = 32). Two of the 3 eligible studies reported the AHI outcomes for 29 patients (42.65%), while all 3 reported O₂ nadir data for the full cohort of 68 patients (100%). Additionally, one study reported OAHI outcomes for 39 patients (57.35%). Among patients with preoperative AHI or OAHI data (n = 47, 69.12%), the majority were classified as having moderate to severe OSA before surgery. Detailed study characteristics are presented in Table 1.

**Table 1.** Study Characteristics.

Author	Year	Number of patients	Mean + SD age of patients	Females (%)	Baseline OAHI/AHI (mild/moderate + severe)	Concurrent surgeries
Prosser	2016	21	9.3 + (4.3)	10 (47.6)	4/17 (AHI)	None
Skirko	2017	39	11 + (4)	16 (41.0)	4/25 (OAHI)	Revision adenoidectomy (9) inferior turbinate reduction, (9) uvulopalatopharyngoplasty (6) epiglottopexy (3) and revision tonsillectomy (11)
Williamson	2024	8	9.86 + (3.71)	6 (75.0%)	3/5 (AHI)	Revision adenotonsillectomy (1) Concurrent supraglottoplasty (6) and one individual underwent both

Abbreviations: AHI, apnea-hypopnea index; NR, not reported; OAHI, obstructive apnea-hypopnea index.

### Pooled Analysis of All Studies

#### Changes in AHI, OAHI Minimum Oxygen Saturation

**Figure 2.**
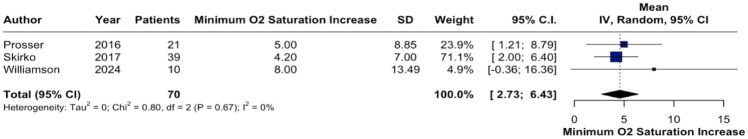
Forest plot of change in the minimum oxygen saturation post lingual tonsillectomy.

**Figure 3.**
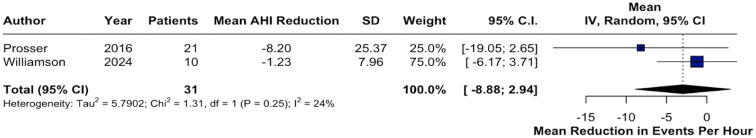
Forest plot of change in the OAHI post lingual tonsillectomy.

**Figure 4.**
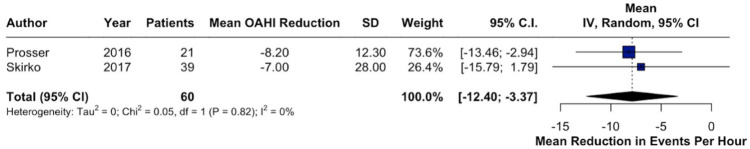
Forest plot of change in the AHI post lingual tonsillectomy.

All 3 studies reported data on the mean change in minimum oxygen saturation among patients undergoing lingual tonsillectomy. Across these studies, O₂ nadir improved by a mean of 4.58% (95% CI: 2.73-6.43, *I*^2^ = 0%; Figure 2). Two studies provided data on both OAHI and AHI,^29,34^ while one study reported exclusively on AHI.^35^ Postoperatively, AHI decreased by −2.97 (95% CI: −8.88 to 2.94, *I*^2^ = 24%; Figure 3), while OAHI demonstrated a more substantial reduction of −8.20 (95% CI: −13.46 to −2.94; *I*^2^ = 0%; Figure 4).

#### Postoperative Complications

Few complications following lingual tonsillectomy were reported (Supplemental Table 2), with notable inconsistencies in reporting across studies. Skirko et al^29^ documented a range of complications, including minor obstruction requiring oxygen support in 28% of patients (n = 11), postoperative vomiting in 3% (n = 1), bleeding with spontaneous resolution not requiring a return to the operating room in 3% (n = 1), and dehydration in 8% (n = 3). In contrast, Williamson et al^35^ reported no complications, despite actively monitoring for adverse events such as postoperative bleeding (with or without OR intervention), poor oral intake, dysphagia, globus sensation, reintubation, and postoperative respiratory infections. Due to the limited number of studies and variability in reported outcomes, a single-arm analysis of these complications could not be performed.

### Quality Assessment

The included non-randomized studies were assessed for risk of bias using the ROBINS-I tool, with individual study appraisal reported in Supplemental Table 1. Overall, Prosser et al^34^ was deemed to be at moderate risk of bias, with Skirko et al^29^ and Williamson et al^35^ determined to be at a serious risk of bias. All studies were downgraded for bias due to confounding factors, as they failed to adequately control for variables such as co-administered surgeries. The overall study number was too low to conduct funnel-plot analysis and Egger’s regression test.

## Discussion

This meta-analysis represents the first evaluation of lingual tonsillectomy in pediatric patients with Down syndrome who have residual OSA following adenotonsillectomy. Our main findings include (i) a significant reduction in AHI and OAHI, (ii) an improvement in O₂ nadir, and (iii) inconsistent reporting of postoperative complications across studies. These findings support the potential efficacy of lingual tonsillectomy in reducing OSA severity in this population, though further standardized reporting is needed to fully understand the risks associated with the procedure.

The increase in minimum oxygen saturation following lingual tonsillectomy was a significant finding, showing an average improvement of 4.58% (95% CI: 2.73-6.43). This result is consistent with observed improvements in oxygen saturation across the broader pediatric population undergoing lingual tonsillectomy as noted in a previous systematic review and meta-analysis, with our study confirming that patients with Down syndrome can experience outcomes, similar to the general pediatric population.^26^ Improved oxygenation is particularly relevant for children with OSA, as chronic hypoxemia can have adverse effects on cognitive and developmental outcomes.^35,36^ In addition to O₂ nadir, our analysis demonstrated significant reductions in both AHI and OAHI following lingual tonsillectomy, with mean decreases of −2.97 (95% CI: −8.88 to 2.94) for AHI and −8.20 (95% CI: −13.46 to −2.94) for OAHI. These improvements indicate a meaningful reduction in apnea and hypopnea events, supporting the notion that lingual tonsillectomy can enhance airway patency during sleep for children with Down syndrome. These same meta-analyses in the general pediatric population with OSA have reported that lingual tonsillectomy can reduce AHI respiratory events, but evidence specific to Down syndrome has been limited. Additionally, we present novel evidence that this procedure also reduces OAHI events. These findings are promising for reducing the burden of OSA symptoms in this high-risk group, suggesting that lingual tonsillectomy may be a valuable addition to the treatment protocol for residual OSA post-adenotonsillectomy.

The question of whether children with Down syndrome experience poorer surgical outcomes following lingual tonsillectomy has been a topic of ongoing investigation.^22^ This is particularly relevant given the increased prevalence of obstructive sleep apnea (OSA) in this population and the complex interplay of anatomical and physiological factors contributing to airway obstruction.^10^ This concern stems from the high likelihood of multiple sites of upper airway obstruction in this population, compounded by contributing factors such as muscular hypotonia, pharyngeal hypotonia, glossoptosis, and relative macroglossia.^37,38^ Although multiple case series have explored these issues,^29,34,35^ no meta-analytical studies have been conducted to comprehensively evaluate these claims. In our study, we observed a lower improvement in oxygen saturation (O₂ sats) and AHI reduction compared to previous pooled analyses. Nonetheless, we found an overall improvement in O₂ saturation and a reduction in AHI and OAHI events. Additionally, we highlight the improved decrease in OAHI, versus AHI. This may indicate that although lingual tonsillectomy removes a structural burden of OSA in children with Down syndrome, there is an additional central component that cannot be ignored. This should be factored into treatment planning, and communication with patients on the expectations for procedure outcomes.

These findings underscore the importance of considering the unique anatomical and physiological characteristics of children with Down syndrome during shared decision-making discussions regarding surgical interventions.

Our findings on postoperative complications were limited by inconsistent reporting across studies. While Skirko et al^29^ detailed minor complications, including airway obstruction requiring oxygen support, postoperative vomiting, and dehydration. Williamson et al^35^ reported no complications despite monitoring for common postoperative issues such as bleeding, dysphagia, and respiratory infections. Overall, in the studies reporting these outcomes, the complication rate was overall low in this procedure. This is reflected in previous studies assessing pediatric patients.^38^ However, given inconsistent reporting between studies, data is limited on the overall morbidity burden of this operation in this subgroup. detailed reporting of adverse events is critical for assessing the safety profile of lingual tonsillectomy, especially in children with Down syndrome, who may be more vulnerable to postoperative complications due to anatomical and physiological predispositions, as confirmed in other procedures.^39^ Future research with standardized complication reporting is essential for guiding clinicians in assessing the risk-benefit profile of this procedure.

This study is not without limitations. The small number of included studies, their retrospective nature, and the variability in outcome reporting introduce the possibility of bias and limit the generalizability of our findings. In addition, the lack of a control group creates difficulties for comparison with other surgical approaches in obstructive sleep apnea. Prospective studies with larger sample sizes, consistent outcome measures, and robust complication reporting are needed to validate our findings and to inform clinical guidelines for managing residual OSA in children with Down syndrome.

## Conclusion

This meta-analysis demonstrates that lingual tonsillectomy significantly improves OSA severity in children with Down syndrome, with reductions in AHI and OAHI and improvements in minimum oxygen saturation. While outcomes are promising, the degree of improvement appears lower compared to the general pediatric population, likely due to anatomical and physiological factors unique to this group. Complications were infrequent, though inconsistently reported, highlighting the need for standardized reporting. Future prospective studies with larger sample sizes are essential to validate these findings and optimize treatment protocols for managing residual OSA in children with Down syndrome.

## Supplemental Material

sj-docx-1-aor-10.1177_00034894251407787 – Supplemental material for Lingual Tonsillectomy for Resistant Obstructive Sleep Apnea in Children with Down Syndrome: A Systematic Review and Meta-Analysis Supplemental material, sj-docx-1-aor-10.1177_00034894251407787 for Lingual Tonsillectomy for Resistant Obstructive Sleep Apnea in Children with Down Syndrome: A Systematic Review and Meta-Analysis by Emma Finnegan, Leticia Campos, Anna Mulcahy and Jaime Doody in Annals of Otology, Rhinology & Laryngology
