# Supplementary material for: Lingual Tonsillectomy for Resistant Obstructive Sleep Apnea in Children with Down Syndrome: A Systematic Review and Meta-Analysis
Source: Ann Otol Rhinol Laryngol. 2026 Jan 3;135(6):429–35. doi: 10.1177/00034894251407787 (PMC13125704; doi:10.1177/00034894251407787)
Supplement: sj-docx-1-aor-10.1177_00034894251407787 – Supplemental material for Lingual Tonsillectomy for Resistant Obstructive Sleep Apnea in Children with Down Syndrome: A Systematic Review and Meta-Analysis [file sj-docx-1-aor-10.1177_00034894251407787.docx]

**SUPPLEMENT:**


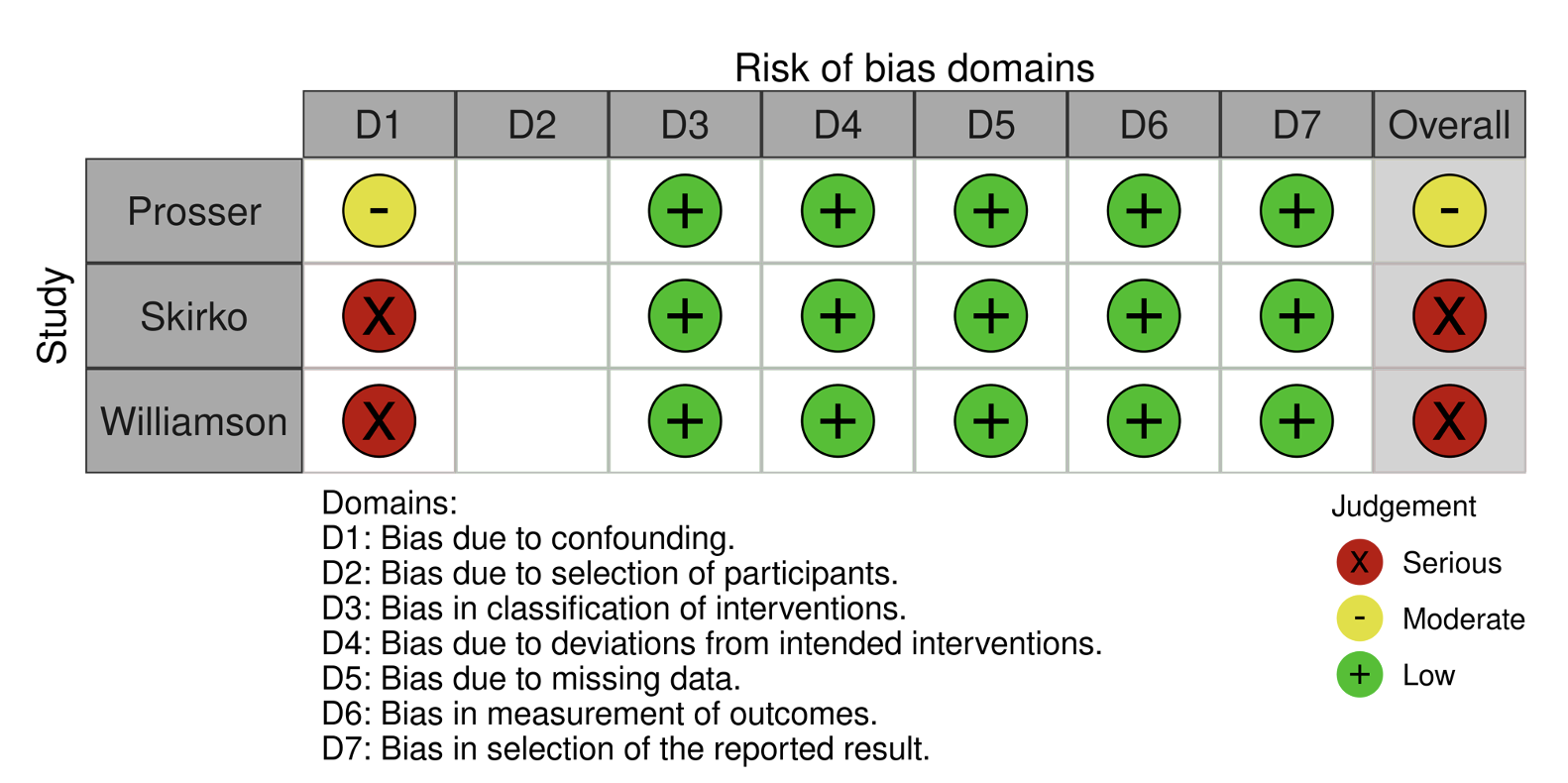


**Supplementary Table 1:** Risk of bias.

| PubMed | 1. (child* OR pediatric OR paediatric) 2. (“lingual tonsillectomy” OR “lingual tonsil”) 3. (OSA OR “obstructive sleep apnea” OR “obstructive sleep apnoea” OR “sleep apnoea” or “sleep apnea” OR “hypopnoea syndrome” OR “sleep apnea syndrome” OR “sleep apnoea syndrome”) 4. #1 AND #2 AND #3 |
| --- | --- |
| Embase | 1. (child* OR 'pediatric'/exp OR pediatric OR 'paediatric'/exp OR paediatric) 2. ('lingual tonsillectomy'/exp OR 'lingual tonsillectomy' OR 'lingual tonsil'/exp OR 'lingual tonsil') 3. (osa OR 'obstructive sleep apnea'/exp OR 'obstructive sleep apnea' OR 'obstructive sleep apnoea'/exp OR 'obstructive sleep apnoea' OR 'sleep apnoea'/exp OR 'sleep apnoea' OR 'sleep apnea'/exp OR 'sleep apnea' OR 'hypopnoea syndrome' OR 'sleep apnea syndrome'/exp OR 'sleep apnea syndrome' OR 'sleep apnoea syndrome'/exp OR 'sleep apnoea syndrome') 4. #1 AND #2 AND #3 |
| Cochrane | 1. (child* OR pediatric OR paediatric) 2. (“lingual tonsillectomy” OR “lingual tonsil”) 3. (OSA OR “obstructive sleep apnea” OR “obstructive sleep apnoea” OR “sleep apnoea” or “sleep apnea” OR “hypopnoea syndrome” OR “sleep apnea syndrome” OR “sleep apnoea syndrome”) 4. #1 AND #2 AND #3 |

**Supplementary Table 2:** Full expanded search strategy

| **Study** | **Year of Publication** | **Complications** |
| --- | --- | --- |
| Prosser | 2018 | Not reported |
| Skirko | 2017 | Minor obstruction requiring oxygen only 11 (28%), post-op vomiting 1 (3%), bleeding not requiring OR 1 (3%), dehydration 3 (8%) |
| Williamson | 2014 | No complications |

**Supplementary Table 3:** Study-reported complications.
